# Multi-Component Vaccine Candidates Against Non-Typeable *Haemophilus influenzae*

**DOI:** 10.3390/vaccines13090892

**Published:** 2025-08-22

**Authors:** Nouria Belkacem, Ala-Eddine Deghmane, Muhamed-Kheir Taha

**Affiliations:** Institut Pasteur, Invasive Bacterial Infections Unit and National Reference Centre for Meningococci and *Haemophilus influenzae*, 75724 Paris Cedex 15, France; ala-eddine.deghmane@pasteur.fr (A.-E.D.); mktaha@pasteur.fr (M.-K.T.)

**Keywords:** NTHi, infection, antibiotic resistance, vaccine, OMP

## Abstract

**Background:** *Haemophilus influenzae* (Hi), a Gram-negative bacterium, is divided into two broad categories: encapsulated and non-capsulated isolates, also called non-typeable Hi isolates (NTHi). NTHi has become prevalent since the introduction of the vaccine against Hi of serotype b. Hi can cause local infections on respiratory mucosal surfaces and urogenital infections, which can lead to septic abortion in pregnant women. It can also cause invasive infections such as meningitis and septicemia. Moreover, NTHi isolates are becoming increasingly resistant to antibiotics. Vaccines targeting NTHi are not yet available. As these NTHi isolates are not encapsulated, vaccines should target proteins at the bacterial surface. However, vaccine development is hindered by the high variability of these proteins. We aimed to identify conserved outer membrane proteins (OMPs) for vaccines against NTHi. **Methods:** We analyzed core-genome multilocus sequence typing (cgMLST) of 1144 genomes of Hi collected between 2017 and 2022 and, of these, identified 514 conserved genes that encoded OMPs. We focused on two specific OMPs: Haem1295, encoding the protein P5 (P5), and Haem1040, encoding the protein 26 (P26). P5 is known to bind human complement regulatory protein factor H (FH), while both P5 and P26 are involved in enhancing immune responses. The genes encoding these proteins were cloned, overexpressed, purified, and tested in both active and passive protection models using systemic infection in mice. **Results:** P5 and P26 were found to be immunogenic during human infections. Vaccination with these proteins conferred protection against both homologous and heterologous NTHi isolates in mice, suggesting broad cross-protection. **Conclusions:** P5 and P26 are promising vaccine candidates showing cross-protection against NTHi and offering the additional benefit of targeting bacterial virulence factors, enhancing vaccine efficacy against NTHi isolates.

## 1. Introduction

*Haemophilus influenzae* (Hi) is a Gram-negative Coccobacillus bacterium commonly found on human mucosal surfaces, particularly in the respiratory tract. Hi can cause local infections on these mucosal surfaces, including acute otitis media, conjunctivitis, sinusitis, and also urogenital infections [1]. Recently, Hi has become the dominant bacterial pathogen in acute otitis media [2].

In addition to local infections, Hi also causes invasive *H. influenzae* diseases (IHiDs) such as septicemia and meningitis [3,4]. Hi isolates are divided into two broad categories: encapsulated (typeable) and non-capsulated (non-typeable) isolates. The capsule structure and antigenicity determine the serotype (designated a to f). Serotype b (Hib) was the most pathogenic in humans, particularly affecting infants and young children. However, Hib infections significantly declined with the introduction of Hib conjugate polysaccharide vaccines [4].

Non-capsulated isolates, also known as “non-typeable” Hi (NTHi), also cause invasive and non-invasive infections in immunocompetent individuals, including meningitis and septicemia [5]. NTHi has also been reported to cause septic abortion upon genital infection in pregnant women [5]. Recent studies suggest that these NTHi isolates are exhibiting increasing virulence and acquisition of antibiotic resistance [6]. Notably, resistance to beta-lactams, including third-generation cephalosporins, poses a significant concern.

The pathogenesis of NTHi, which lacks the polysaccharide capsule, is multifactorial and involves various virulence factors such as lipooligosaccharides (LOS), adhesins, and outer membrane proteins (OMP). Biofilm formation has also been implicated in chronic and recurrent respiratory infections [7]. In contrast to typeable isolates, which are relatively more genetically homogeneous, NTHi isolates demonstrate considerable genetic diversity, which represents an important hallmark and a major obstacle that makes the development of successful vaccines an unmet need [6,7]. Oral vaccination using whole-cell NTHi by oral vaccination does not yield a significant reduction in the number and severity of exacerbations [8]. Although protein D from *H. influenzae* is incorporated as a carrier protein in the licensed 10-valent pneumococcal conjugate vaccine, robust evidence supporting its protective efficacy against NTHi otitis has yet to be established [9].

The availability of whole-genome sequencing (WGS) data from large collections of Hi isolates, including NTHi, has the potential to identify vaccine candidates against NTHi. The increasing antibiotic resistance of NTHi constitutes an emerging public health problem. The lack of a preventive strategy for NTHi infections represents a medical need that we aim to address by exploring vaccine candidates against these isolates. In this study, we utilized a recent large collection of Hi isolates to identify potential vaccines. We aimed to investigate the ability of these candidates to promote protection against systemic experimental infection in an animal model and to assess their immunogenicity during natural infection in humans. Additionally, we sought to determine the breadth of coverage of NTHi isolates by antibodies against these vaccine candidates.

## 2. Materials and Methods

### 2.1. H. influenzae and Growth Conditions

Hi isolates were sent to the National Reference Centre for *meningococci* and *Haemophilus influenzae* (NRCMHi) at the Institut Pasteur as part of its mission of surveillance [10]. Isolates were cultured in Chocolate + PolyViteX agar plates (bioMerieux, Craponne, France) at 37 °C in the presence of 5% CO_2_.

### 2.2. WGS, Bioinformatics Analysis and Selection of Vaccine Candidates

WGS was performed on Hi isolates, as previously described [10]. Data were analyzed using available tools on the PUBMLST.org platform including core-genome MLST using Grape Tree and the presence/absence tool [11]. CLUSTAL 2.1 Multiple Sequence Alignments (https://www.genome.jp/tools-bin/clustalw) (accessed on 3 September 2024) was used to align the deduced amino acid (aa) sequences of P5 (247–361 aa, 36 kDa), and P26 (197 aa, 26 kDa) proteins to perform phylogenetic analysis. The distance matrix obtained was analyzed by SplitsTree4 [12].

### 2.3. Subcloning, Expression and Purification of P5 and P26

DNA fragments encoding the P5 open reading frame (allele 302 and allele 12) were amplified from NTHi DNA by PCR (isolates LNP31258 and LNP32433, respectively) using specific primers:

Nco_P5fw: AAACCATGGCTCCACAAGAAAACACTTT.

Xho_P5rev: GCCCTCGAGTTTAGTACCGTTTACCGCGA.

DNA fragments encoding the P26 (allele 3) open reading frame were amplified from the NTHi isolate LNP31429 using specific primers:

Nco_OMP26fw: GGTCTCCCATGG AAGAAAAAAT TGCTTTCATT.

Xho_OMP26rev: GCCCTCGAGTTTTTTCTCTTGTGCTTTTTC.

Both forward and reverse primers were carrying, respectively, the NcoI and XhoI restriction sites (underlined sequences) as adaptors to facilitate the subcloning. The PCR amplicons lacking the N-terminal signal peptide-encoding sequences were digested with NcoI and XhoI and inserted into the pET28b vector (Novagen, Madison, WI, USA) between NcoI and XhoI. The resulting recombinant plasmids carried P5 (pET-P5-302 and pET-P5-12 plasmids)- or P26 (pET-P26-3 plasmid)-encoding genes in frame with coding sequences for a C-terminal hexahistidine tag.

The recombination plasmids or pET28b empty vector were transformed into *E. coli* BL21 (DE3) strain (Novagen, USA), as previously described [13]. Transformants were cultured at 37 °C in Luria–Bertani (LB) medium (Sigma Adrich, Saint-Quentin-Fallavier, France) in the presence of kanamycin (50 mg/mL) and chloramphenicol (20 mg/mL) with shaking, until the optical density at 600 nm reached 0.6–0.7, when proteins were then overproduced by induction with isopropyl β-D-thiogalactopyranoside (1 mM) for 2H. Cells were harvested by centrifugation at 10,000× rpm at 4 °C for 15 min, and resuspended with buffer (50 mM NaH_2_PO_4_, 300 mM NaCl, pH 8.0), followed by sonication. The sonicated culture was centrifuged (10,000× rpm, 15 min, 4 °C) and proteins were purified from the supernatant, using a Ni-NTA resin (QIAGEN, Courtaboeuf, France) according to the manufacturer’s instructions.

Proteins were eluted using different concentrations of imidazole (25 mM, 50 mM, 100 mM or 200 mM) in 50 mM NaH_2_PO_4_, 300 mM NaCl, pH 8.0. Imidazole was then removed by dialyzing the eluates (containing purified proteins) 3 times with 1 L of buffer (50 mM NaH_2_PO_4_, 300 mM NaCl, pH 8.0) for 3 h, at 4 °C. Expression of the proteins was assessed by SDS-PAGE after protein separation using 4–12% Bis-Tris acrylamide gel, followed by Coomassie staining or Western immunoblot using monoclonal HRP-conjugated anti-histidine tag antibody (Abcam, Cambridge, UK). Protein concentrations were determined with the nanodrop according to the manufacturer’s instructions.

### 2.4. Immunogenicity of Vaccine Candidates During Natural Infection

The purified proteins (P5, either P5-302 or P5-12, and P26-3) and the whole bacterial extracts of different Hi serotypes were used to analyze their immunogenicity during natural infections by Western blotting using sera from a patient with confirmed Hib infection. The interaction was detected using a secondary HRP-conjugated anti-human IgG antibody (ThermoFisher, IllKrich, France).

### 2.5. Ethical Statement

Animal work in this study was carried out at the Institut Pasteur in strict accordance with the European Union Directive 2010/63/EU (and its revision 86/609/EEC) on the protection of animals used for scientific purposes. The protocol was approved by the Institut Pasteur Review Board that is part of the Regional Committee of Ethics of Animal Experiments of Paris Region (Permit Number 75-1554) and the approval of the Institut Pasteur Board (Dap210087/N°APAFIS #34085-2021112212171298).

### 2.6. Mice

To study *H. influenzae* infection, we used the animal model of double transgenic mice expressing human transferrin (as an iron source for Haemophilus growth) and human factor H (fH) [14], a negative regulator of the complement pathway that binds to the bacterial surface, thereby allowing *H. influenzae* to escape complement-mediated lysis. Mice were bred in-house and housed in a biosafety containment facility in filtered cages with sterile bedding, water, and food, according to institutional guidelines.

### 2.7. Experimental Active and Passive Protection of Vaccine Candidates in Mice

Active protection was provided by immunizing mice with the purified proteins. Four groups of mice (*n * =  10 per group) on days 0, 7, and 21 were injected via the intra-peritoneal route. Vaccine combinations included P5 alone (12.5 µg/mouse of each protein P5-12 and P5-302), P26 alone (25 µg/mouse), and a mixture of P5 (including both variants P5-12 and P5-302) with P26 (25 µg of each protein/mouse), and the unimmunized mice were injected with the same volume of phosphate-buffered saline (PBS). Serum samples were taken before immunization and at day 28 after the first injection to evaluate the immune response by Western blotting.

Sera collected from mice immunized by active protection were used in passive protection. Identical groups of mice were used and injected intravenously with 2 µg of total IgG per mouse and per immunizing protein. For the control group, mice were injected with sera obtained from unvaccinated mice.

Mice were challenged intra-peritoneally on day 35 for active protection and at 24 h after intravenous injection for passive protection with an NTHi isolate (LNP31258) (10^9^ cfu/mouse). Bacterial loads were evaluated in blood samples collected at 2 h, 6 h, and 24 h after infection.

### 2.8. Responses in Mice to Vaccine Candidates

Peritoneal washes from mice were performed by injecting and recovering 2 mL of physiological water. Cells from washes were first incubated with Fc block (anti-CD16/32) for 10 min on ice, followed by staining with fluorochrome-conjugated primary antibodies for 30 min in the dark at 4 °C. Antibodies used included MHCII, CD45, F4/80, CD11b, Ly6G, CD11b, CD11c, MHC-II, and Ly6C. Data were acquired on LSRFortessa and analyzed using FlowJo software (version 10.10.0). Cell population frequencies and absolute numbers were compared between vaccinated and control groups using GraphPad Prism software, version 10. Data are presented as mean ± SEM.

Enzyme-linked immunosorbent assays (ELISAs) were also performed on dilutions of individual serum samples from vaccinated and unvaccinated mice to detect IgG. The ELISA plates were coated with 0.1 mL of 5 µg/mL of purified proteins P5-302, P5-12 or P26-3. ELISAs used secondary antibodies conjugated to horseradish peroxidase (HRP) through a chemiluminescent reaction, as previously described [15].

### 2.9. Statistical Analysis

The results are shown as the mean ± SEM. The statistical significance of differences between two samples was evaluated using Student’s *t*-test; *p* < 0.05 was considered statistically significant.

## 3. Results

### 3.1. Rationale Behind the Selection of Genes Encoding Proteins Included as HiNT Vaccine Candidates

**Figure 1:**
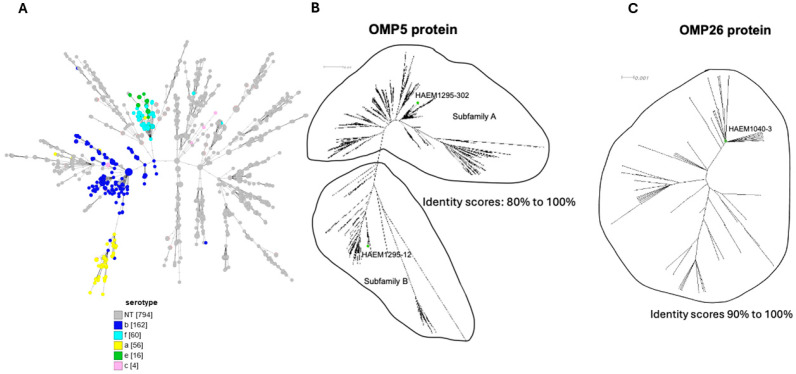
(**A**) Gene-by-gene GrapeTree software version 10 representation based on core-genome multilocus sequence typing of the 1092 isolates. These genetic relationships were explored using BIGSdb tools available on the PubMLST.org site. The different serotypes and HiNT are indicated with the different colors. The numbers of each serotype or non-typeable isolates are indicated with the numbers in brackets. (**B**,**C**) Neighbor-joining tree generated based on the ClustalW alignment of 1121 identified P5 protein and 197 identified P26 protein sequences. CLUSTAL 2.1 Multiple Sequence Alignments (https://www.genome.jp/tools-bin/clustalw accessed on 19 August 2025) was used to align the deduced amino acid (aa) sequences of P5 (247–361 aa, 36 kDa) (**B**), and P26 (197 aa, 26 kDa) (**C**) to perform phylogenetic analysis. The distance matrix obtained was analyzed by SplitsTree4. The phylogenetic trees allowed for the selection of three proteins for further analysis (two variants of P5 and one variant of P26), indicated by green circles.

Among all of the Hi isolates received in our laboratory for epidemiological surveillance between 2017 and 2022, 1144 Hi isolates, for which clinical data were available, were selected. WGS was available for 1092 isolates. These sequences were uploaded to the PUBMLST.org platform. These isolates belonged to different serotypes as well as NTHi. Genomic data were analyzed by cgMLST, which showed high diversity in particular for NTHi isolates, unlike the serotypeable isolates, which clustered together for each serotype (Figure 1A). We first used the ‘presence/absence’ gene tool on PubMLST.org that screens each tested genome for the presence or absence of a given gene regardless of the homology level. This analysis identified 514 conserved genes across the 1144 isolates. From these, we focused on 13 genes known to encode for membrane proteins, two of them, in particular, encoding outer membrane proteins: Haem1295, which encodes for the outer membrane protein P5 (P5), and Haem1040, which encodes for the outer membrane protein P26 (P26).

We aligned the deduced amino acid sequences of all alleles available for these two genes using CLUSTAL 2.1 (Multiple Sequence Alignments, to perform phylogenetic analysis). The resulting distance matrix was analyzed using SplitsTree4 (as described in the Methods Section) based on the phylogenetic diversity (PD), which is the sum of the weights for all splits. All of the P5 protein sequences fell into one of two non-empty groups referred to as Subfamily A and Subfamily B (Figure 1B).

The total PD for all of the described P5 proteins was 6.3. PD values for families A and B were 4.3 and 1.9, respectively. Due to the high PD within the P5 protein families, we selected one representative P5 protein among the top 15 most frequent alleles within each subfamily. Accordingly, alleles encoding P5-12 (subfamily B) and P5-302 (subfamily A) were selected, subcloned, and overexpressed in *E. coli* to produce sufficient amounts of soluble protein (Supplementary Tables S1 and S2).

In order to increase the vaccine coverage for the NTHi isolates, we added another conserved protein, P26 (Haem1040), which is less variable than the P5 protein with PD at 0.32. We selected one allele, allele 3, which is the most frequent (P26-3) and that was also subcloned and overexpressed in *E. coli* (Figure 1C).

### 3.2. Immunogenicity of Vaccine Candidates During Natural Infection

**Figure 2:**
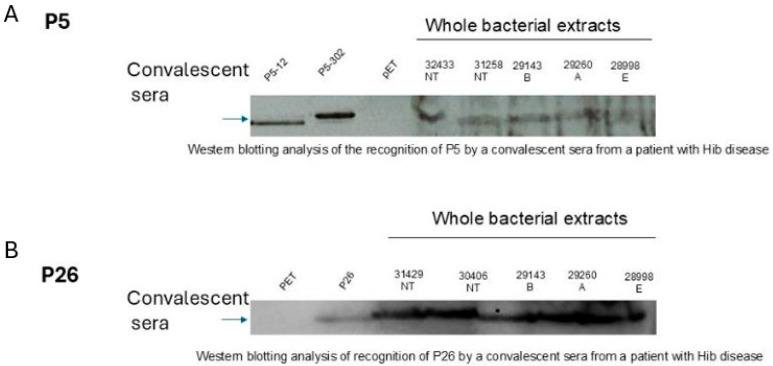
Immunogenicity of vaccine candidates P5/P26 during natural infection. A representative experiment of Western blot analysis showing the recognition of P5 (**A**) and P26 (**B**) from total bacterial lysates of different Hi isolates, NT: non-typeable. B, A, and E stand for *H. influenzae* of serotypes b (Hib), a (Hia), and e (Hie), from convalescent serum from a patient with confirmed Hi disease. Purified proteins P5-12, P5-302, and P26 were used as positive controls and bacterial lysate from empty vector pET28b-transformed *E. coli* (pET) was used as a negative control.

Convalescent sera from patients recovering from Hi invasive infections were used in Western blotting in order to study the immunogenicity of our vaccine candidates during natural infection. Reactive bands of apparent molecular weight of 36 kDa, corresponding to the P5 protein, and those of 26 kDa, corresponding to the P26 protein (Figure 2), were detected from whole bacterial extracts of different serotypes or HiNT isolates as well as purified proteins (Figure 2). These data suggest that P5 and P26 are expressed and immunogenic during invasive Hi infections.

### 3.3. Experimental Active and Passive Protection of Vaccine Candidates in Mice

Serum samples were collected before immunization and on day 28 after the first injection. Western blotting was performed to assess the immune response, comparing immunized mice with control mice. The sera from all of the vaccinated mice reacted with the corresponding recombinant protein, while no reactivity was observed for the sera from the unvaccinated control mice.

**Figure 3:**
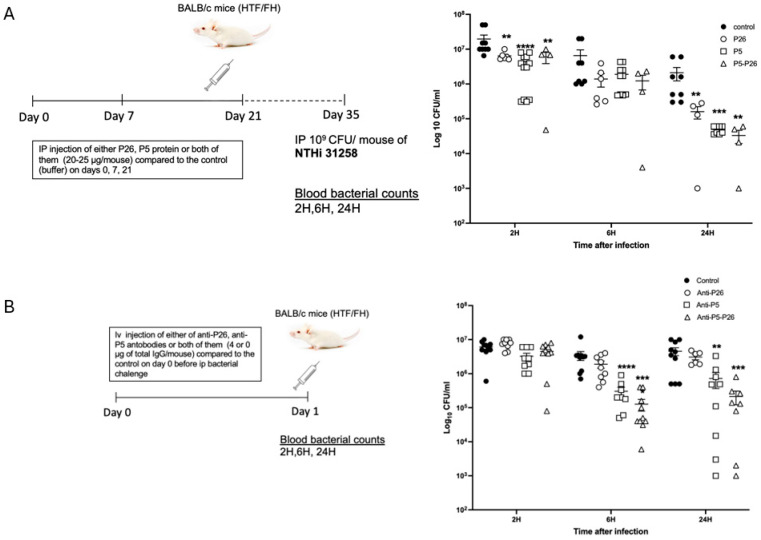
(**A**) The schedule for immunization and experimental design for evaluation of the active protection of mice with P5 and P26. Active protection of mice was provided by immunizing mice with the purified proteins via the intra-peritoneal route (on days; 0, 7 and 21). Four groups were used for each type of immunization: vaccination using P5 (12.5 µg/mouse of each protein P5-12 and P5-302), vaccination using P26 (25 µg/mouse), vaccination using both P5 (12.5 µg/mouse for each protein P5-12 and P5-302) and P26 (25 µg/mouse), and unvaccinated control mice. Mice were challenged intra-peritoneally on day 35 with an NTHi isolate (LNP31258) (10^9^ cfu/mouse). Bacterial counts in blood were determined at 2, 6 and 24 h after infection. Results are expressed as mean ± SEM, the level of the significant difference between serogroups are indicated as follows (** *p* < 0.01, *** *p* < 0.001). (**B**) The schedule for immunization and experimental design for evaluation of the passive protection of mice with P5 and P26. Passive protection was performed by injecting sera from immunized mice into naïve mice intravenously. The injection was performed with 2 µg of total IgG per mouse and per immunizing protein. Control mice were injected with serum from non-immunized mice (control mice from the active immunization). Mice were challenged via the intra-peritoneal route 12 h later with an NTHi isolate LNP31258 (10^9^ cfu/mouse). Bacterial counts in the blood of each group were determined at 2, 6 and 24 h after infection. Results are expressed as mean ± SEM, the level of the significant difference between serogroups are indicated as follows (** *p* < 0.01, *** *p* < 0.001, **** *p* < 0.0001).

Thereafter, we challenged the vaccinated and unvaccinated mice via the intra-peritoneal route on day 35 after the first immunization. The mice were infected with the NTHi isolate LNP31258 (expressing P5-302) (10^9^ cfu/mouse) (Figure 3A).

The bacteria in the blood were cleared significantly faster in the mice immunized with either the P5 or P26 protein when compared to the unvaccinated control mice, after 2 h and 24 h of infection. Interestingly, synergistic clearance was observed in the mice immunized with a mixture of both P5 and P26, with significant differences at 24 h post-infection (Figure 3A).

We next tested passive protection using identical groups of mice that were injected intravenously with 2 µg of total IgG per mouse and per immunizing protein. The mice were challenged via the intra-peritoneal route 24 h after a passive injection of antibodies with the NTHi isolate (LNP31258) (10^9^ cfu/mouse) (Figure 3B).

As for active immunization, our data showed faster clearance of bacteria from the blood of mice that received anti-P5 or anti-P26 immune sera compared to the control mice. Significantly faster clearance was observed at 6 h and 24 h after infection in the anti-P5 injected group compared to the control group. Interestingly, a synergistic effect was observed in the mice that received both anti-P5 and anti-P26, with significant differences 6 h and 24 h after infections (Figure 3B).

### 3.4. Responses in Mice to Vaccine Candidates

**Figure 4:**
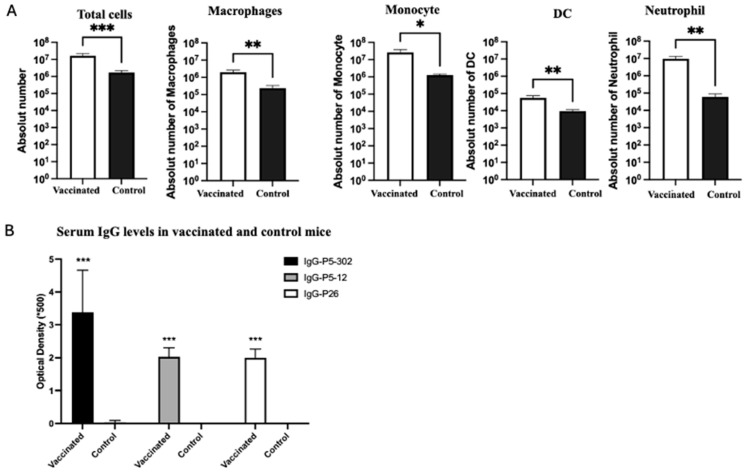
(**A**) Flow cytometry analysis of the impact of vaccination on the immune response in the peritoneal cavity compared to unvaccinated group. Results are presented as absolute numbers and expressed as mean ± SEM, the level of the significant difference between serogroups are indicated as follows (* *p* < 0.05, ** *p* < 0.01, *** *p* < 0.001). DC: dendritic cells. (**B**) IgG levels in the sera of vaccinated mice compared to control unvaccinated mice, measured by ELISA, against each of the purified proteins: P5-302, P5-12, and P26. Results are expressed as mean ± SEM, the level of the significant difference between serogroups are indicated as follows (*** *p* < 0.001).

The immune cell populations in the peritoneal cavity were assessed by flow cytometry 24 h after NTHi infection in both the vaccinated and unvaccinated mice. Our results demonstrate the enhanced recruitment of immune cells to the peritoneal cavity compared to that in the control group (Figure 4A). These recruited immune cells included macrophages, monocytes, dendritic cells (DCs), and neutrophils.

The IgG levels in the sera of the vaccinated and unvaccinated mice were also measured by ELISA. Our results showed that vaccination significantly induced a higher IgG response in the blood of the vaccinated group compared to the control group against each of the purified proteins: P5-302, P5-12, and P26. Notably, the highest IgG level was observed against the purified P5-302 protein (Figure 4B).

### 3.5. Cross-Reactivity of Mice Sera with Proteins Expressed in Diverse Isolates

The polyclonal anti-P5 and anti-P26 antibodies obtained from the active protection of the mice were used in a WB analysis in order to study the cross-reactivities with other P5 and P26 proteins harbored by different isolates of Hi (typeable and non-typeable Hi).

**Figure 5:**
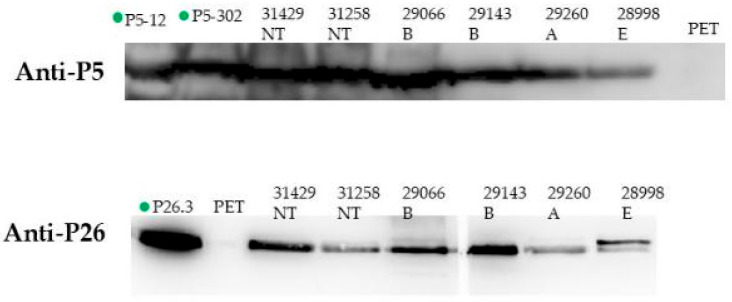
Western blot analysis showing the cross-reactivity of anti-P5 and anti-P26 sera with proteins expressed in different isolates with other P5 and P26 proteins harbored by different isolates of Hi (typeable and non-typeable Hi). The green circles represent the purified proteins P5 and P26 (positive controls). The empty vector (PET) was used as a negative control. NT: non-typeable. B, A, and E stand for *H. influenzae* of serotypes b (Hib), a (Hia), and e (Hie).

Our results showed that the anti-P5 antibodies either for allele 302 or 12 cross-react with the P5 protein belonging to both subfamilies A and B harbored by different Hi isolates. Similar results were observed with the anti-P26 antibody that cross-reacts with different alleles of the P26 protein harbored by different isolates (Figure 5).

### 3.6. Breadth of Coverage During Experimental Infection in Mice

**Figure 6:**
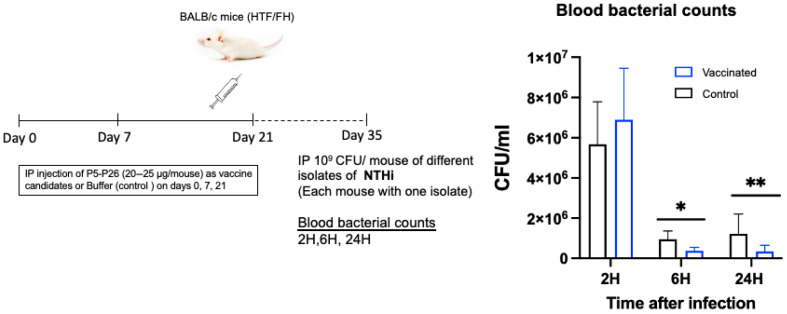
The schedule for active immunization for evaluation of cross-protection of mice with P5 and P26 against different NTHi isolates. Active protection was provided by immunizing mice via the intra-peritoneal route with the purified proteins P5 (12.5 µg/mouse for each protein P5-12 and P5-302) and P26 (25 µg/mouse) or with the buffer as control. The immunization was performed through three doses at day 0, 7, and 21. Mice were challenged via the intra-peritoneal route on day 35 with NTHi isolates expressing different P5 and P26 alleles (10^9^ cfu/mouse) listed in Supplementary Table S3. Each mouse was challenged with one isolate. Bacterial counts in the blood samples of each group were determined at 2, 6, and 24 h after infection by plating on chocolate agar plates. Data are expressed as colony-forming unit per mL (CFU/mL). Results are expressed as mean ± SEM (* *p* < 0.05, ** *p* < 0.01 Student’s *t*-test).

A collection of several NTHi isolates of different genotypes (based on WGS), listed in Supplementary Table S3, were used to assess the breadth of isolate cross-protection during experimental infection in mice immunized with a mixture of P5-12, P5-302, and P26-3 proteins. Coverage was tested in actively immunized mice as described above (Figure 6).

Our results showed that although a higher bacteremia level was observed in the vaccinated group at 2 h after infection compared to the control, significantly lower levels of bacteremia were observed in the vaccinated group compared to the control at 6 h and 24 h after infection. These results suggest faster clearance of bacteria expressing heterogenous alleles of P5 or P26 encoding genes in vaccinated mice. The rapid clearance of capsulated (typeable) isolates was also observed in the immunized mice, but the difference did not reach a significant level, most likely due to the presence of the capsule (Supplementary Figure S1).

## 4. Discussion

The pathogenesis of NTHi, which lacks the polysaccharide capsule, is multifactorial and associated with multiple virulence factors such as lipooligosaccharide (LOS), adhesins, and outer membrane proteins (OMP). Biofilm formation is also implicated in chronic and recurrent respiratory tract infections [7,16,17]. “Heterogeneity” is a hallmark of NTHi and, so far, has been a major obstacle in the development of a successful vaccine. However, several studies have been conducted to identify potential vaccine candidates. Several works highlighted P5 and P26 as potential candidates. P5 and P26 were suggested in the composition of vaccines against NTHi [18,19]. Anti-P5 antibodies were also reported in sera collected from children with acute otitis media (AOM) due to NTHi [20]. However, the vaccine composition may have been hindered by the lack of tools in the pre-genomic era to evaluate the antigenic variability of potential vaccine antigens in NTHi isolates. Most of these studies were limited to non-systematically selected isolates and did not allow for the breadth of coverage of NTHi isolates to be tested [19]. The availability of WGS data from large collections of Hi isolates, including NTHi, allowed for a systemic approach in identifying vaccine candidates against NTHi (large collection of 1144 Hi isolates from 2017 to 2022). Our analysis identified several conserved proteins as vaccine candidates that are also involved in the virulence of Hi. P5 is a surface-exposed protein with a high level of expression (P5 is a homolog of the OmpA family of outer membrane proteins that have a high copy number in the outer membrane of mainly Gram-negative bacteria) [21]. It contributes to the stability of the outer membrane protein. It is also involved in bacterial pathogenesis: P5 was shown to directly bind human complement regulatory protein factor H (fH), thereby reducing complement factor C3 deposition on the bacterial surface and enhancing bacterial survival [22].

Using P5 as a vaccine candidate is hitting two targets with one shot: a protective immune response and a reduction in bacterial virulence. Our data support this hypothesis. Not only are anti-P5 sera protective in mice but anti-P5 sera also significantly reduce fH binding and thus enhance complement activation on bacteria.

The P5 protein seems to be heterogeneous, and we identified two families of P5 proteins. Two variants, one from each subfamily, was included to increase the strain coverage. These variants (P5-12 and P5-302) were chosen according to their frequency among isolates of each subfamily, their association with invasive isolates, and their ability to be subcloned and overexpressed in *Escherichia coli*, generating sufficient amounts of soluble protein.

Immunization studies using each protein individually in mice show significant potential for these proteins to eliminate their homologous isolate efficiently.

Further, adding the P26-3 protein is also expected to increase the breadth of stain coverage. P26 is a highly conserved protein across NTHi isolates with identity scores ranging between 90% and 100%. P26 induces protective responses in animal models and has been shown to activate T and B cells [23]. The combination of the two proteins allowed for rapid bacterial clearance in immunized mice even with a high bacterial load. Our data also suggest that including both P5 and P26 variants in the immunization process produces a synergistic effect in mice, as observed in both active and passive immunizations within a systemic infection model. Our results demonstrated that the combination of P5 and P26 provided enhanced protection compared to each protein administered individually. Our results also suggest that no antigenic competition occurred between the proteins (P5 and P26) in our model, as observed in another study between protein D and OMP26 of Hi [24]. A cooperative/synergistic interplay of antibodies with different antigen recognition specificities in tackling Hi infection may significantly enhance the possibility of achieving protection by activating the complement pathway even in the presence of suboptimal levels of antigen expression. This would overcome the threshold level required for killing. On the other hand, immunization with P5 elicits antibodies that neutralize binding to the human complement regulatory protein factor H (FH). These antibodies that are neutralizing in function may enhance the efficiency of bactericidal antibodies activated by the P26 protein.

Our data also provide evidence of protection against systemic infection with NTHi, which is the most frequently reported *H. influenzae* isolate in invasive infections with this bacterium in Europe [25].

Our data suggest that the synergistic effect elicited by P5 and P26 in immunized mice may be attributed to the significantly higher recruitment of immune cells into the peritoneal cavity in vaccinated groups compared to controls. These immune cells include macrophages, monocytes, dendritic cells, and neutrophils, which contribute to more efficient bacterial clearance. Our data pave the way for further studies on the use of these two proteins (P5 and P26) in a multi-component protein-based vaccine against NTHi and to evaluate the protective effect against local infections such as respiratory infections [26].

## 5. Patents

Patent application: EP 25305565.1. Multi-components vaccine candidates against Non-typeable *Haemophilus influenzae*.

## Data Availability

The original contributions presented in this study are included in the article and sequences are accessible on the PUBMLST.org platform using the IDs provided in the Supplementary Table S3. If required, additional information can be requested from the corresponding author.

## Supplementary Materials

The following supporting information can be downloaded at https://www.mdpi.com/article/10.3390/vaccines13090892/s1, Figure S1: Passive protection against a clinical Hib isolate. Figure S2: A representative experiment of the whole Western blot analysis showing the recognition of P5 (A) and P26 (B) from total bacterial lysates of different Hi isolates by convalescent serum from a patient with confirmed Hi disease. Figure S3: A representative experiment of the whole Western blot analysis showing the cross-reactivity of anti-P5 and anti-P26 sera with proteins expressed in different isolates of Hi (Typeable and non-typeable Hi). Table S1: Representative selected P5-12 proteins among the top 15 most frequent alleles within subfamily B. Table S2: Representative selected P5-302 proteins among the top 15 most frequent alleles within subfamily A. Table S3: NTHi isolates used to challenge vaccinated and unvaccinated mice.
