# Supplementary material for: Multi-Component Vaccine Candidates Against Non-Typeable Haemophilus influenzae
Source: Vaccines (Basel). 2025 Aug 22;13(9):892. doi: 10.3390/vaccines13090892 (PMC12474234; doi:10.3390/vaccines13090892)
Supplement: Supplementary file 1 [file vaccines-13-00892-s001.zip › manuscript-supplementary/SuppFig1.pdf]

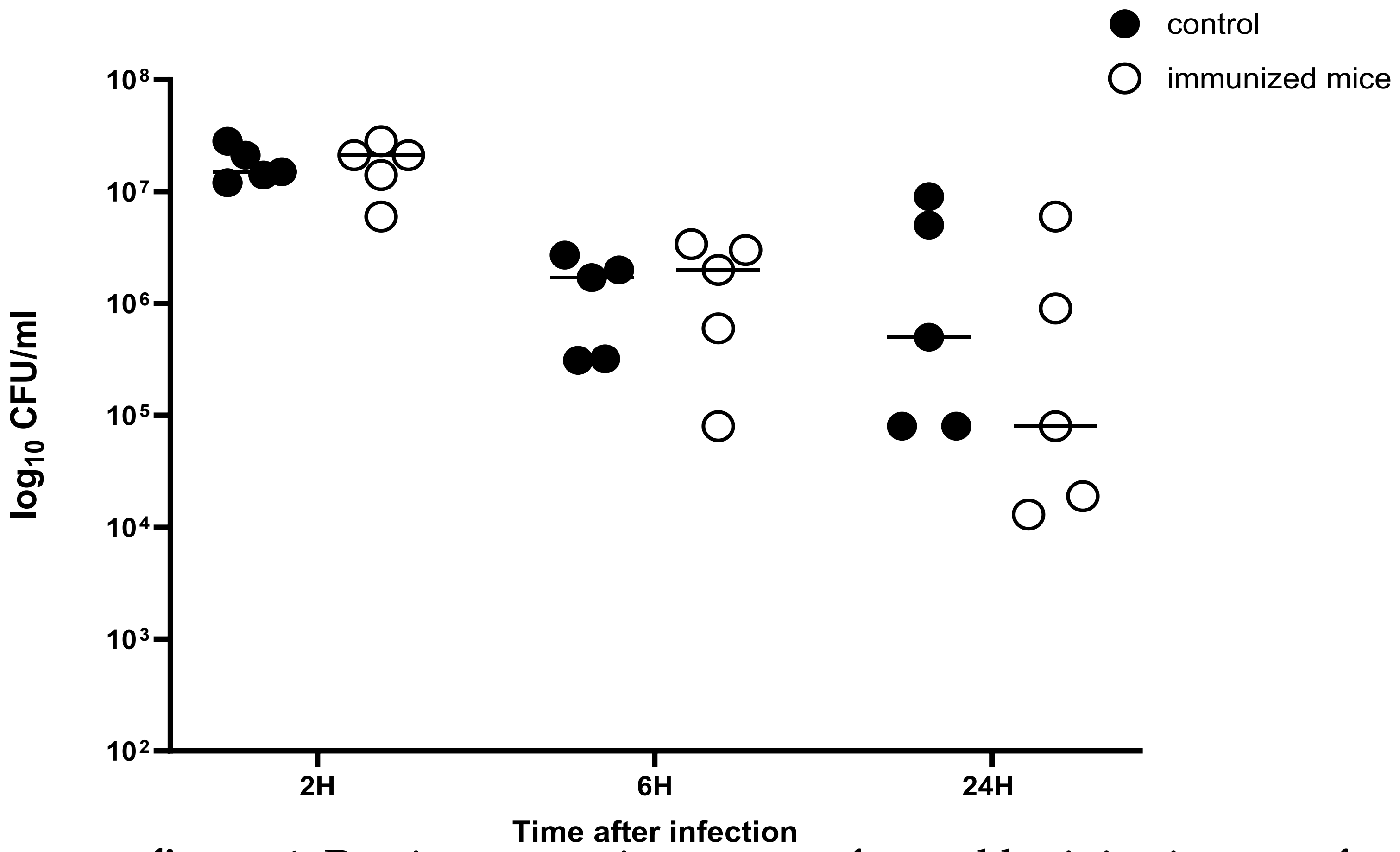

Supplementary figure 1. Passive protection was performed by injecting sera from immunized mice into naïve mice intravenously. The injection was performed with 2 µg of total IgG per mouse and per immunizing protein. Control mice were injected with serum from non-immunized mice (control mice from the active immunization). Mice were challenged in intra-peritoneal route 12h later, with a clinical Hib isolate (10^9 cfu/mouse). Bacterial counts in the blood of each group were determined at 2, 6 and 24h after infection. Results are expressed as mean ± SEM
