# Supplementary material for: Multi-Component Vaccine Candidates Against Non-Typeable Haemophilus influenzae
Source: Vaccines (Basel). 2025 Aug 22;13(9):892. doi: 10.3390/vaccines13090892 (PMC12474234; doi:10.3390/vaccines13090892)
Supplement: Supplementary file 1 [file vaccines-13-00892-s001.zip › manuscript-supplementary/SuppFig.3.pdf]

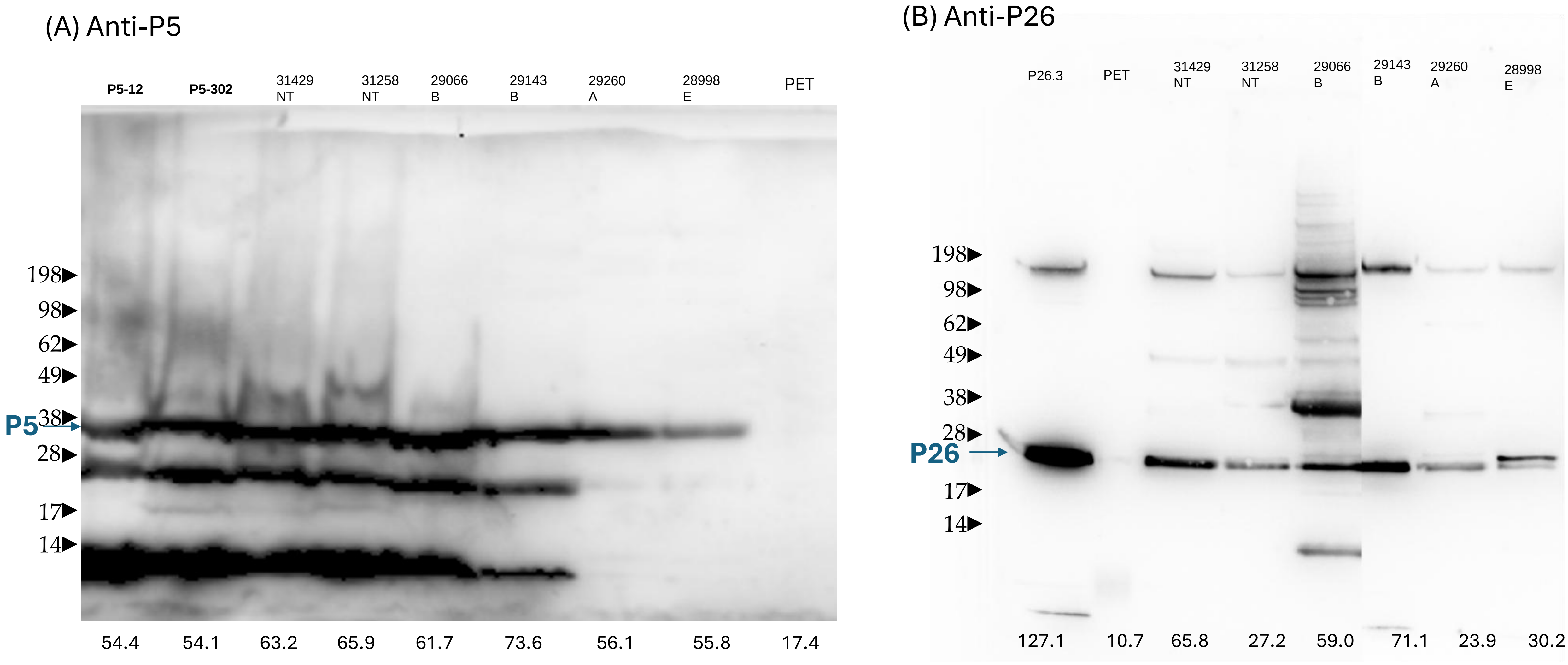

Supplementary figure 3: A representative experiment of the whole Western blot analysis showing the cross-reactivity of anti-P5 and anti-P26 sera with proteins expressed in different isolates of Hi (Typeable and non-typeable Hi). The purified proteins P5 and P26 (positive controls) are indicated. The empty vector (PET) was used as a negative control. NT: non typeable. B, A and E stand for *H. influenzae* of serotypes b (Hib), a (Hia) and e (Hie). The positions of P5 and P26 are indicated by the bleu arrows. The numbers under each photo indicate densitometry readings/intensity ratios of each band of P5 and P26 using imageJ 1.51k (https://imagej.net/ij/). Molecular weight markers (Kda) are indicated by black arrowheads.
