# Supplementary material for: Multi-Component Vaccine Candidates Against Non-Typeable Haemophilus influenzae
Source: Vaccines (Basel). 2025 Aug 22;13(9):892. doi: 10.3390/vaccines13090892 (PMC12474234; doi:10.3390/vaccines13090892)
Supplement: Supplementary file 1 [file vaccines-13-00892-s001.zip › manuscript-supplementary/SuppFig.2.pdf]

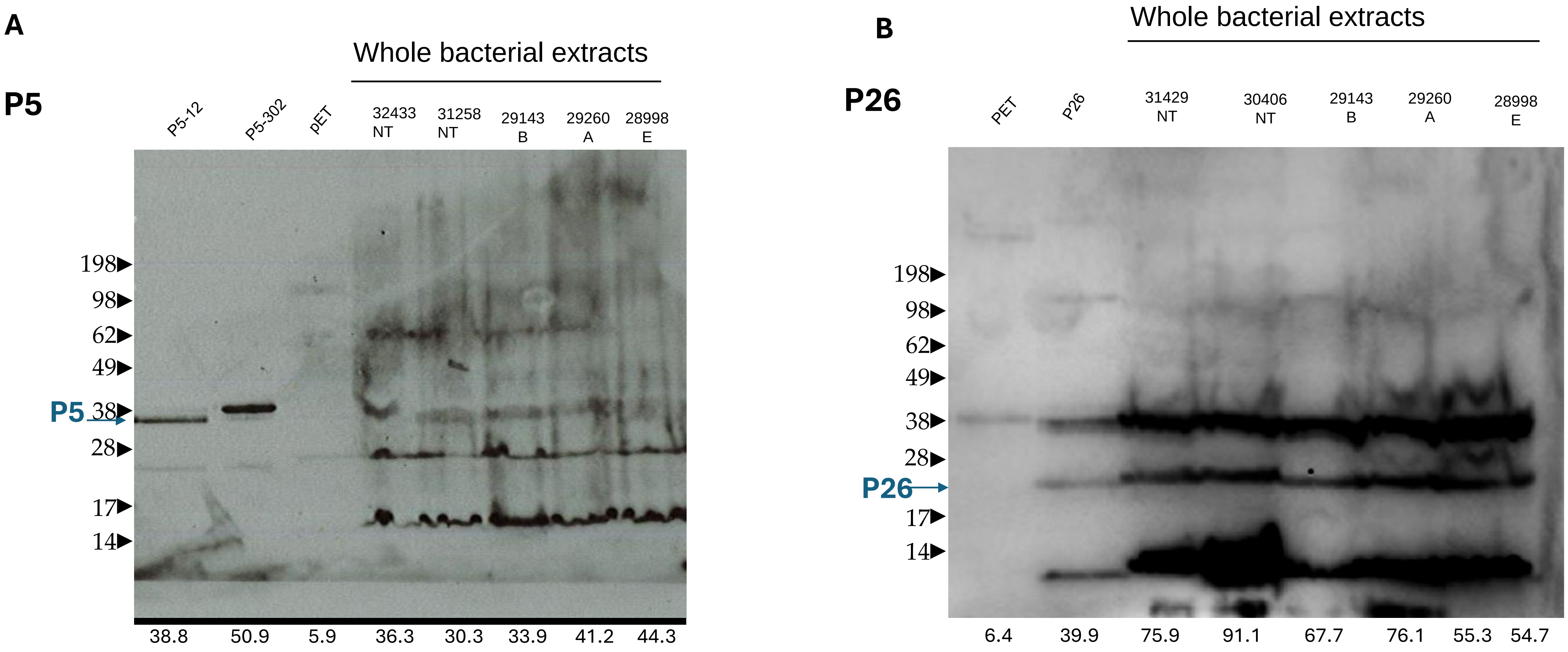

Supplementary figure 2: A representative experiment of the whole Western blot analysis showing the recognition of P5 (A) and P26 (B) from total bacterial lysates of different Hi isolates by convalescent serum from a patient with confirmed Hi disease. Purified proteins P5-12, P5-302 and P26 were used ass positive controls and bacterial lysate from empty vector pET28b-transfomred *E. coli* (pET) was used as a negative control. The positions of P5 and P26 are indicated by the arrows. The numbers under each photo indicate densitometry readings/intensity ratios of each band of P5 and P26 using imageJ 1.51k (https://imagej.net/ij/). Molecular weight markers (Kda) are indicated by black arrowheads.
