# Supplementary figures and images for: Multi-Component Vaccine Candidates Against Non-Typeable Haemophilus influenzae
Source: Vaccines (Basel). 2025 Aug 22;13(9):892. doi: 10.3390/vaccines13090892 (PMC12474234; doi:10.3390/vaccines13090892)

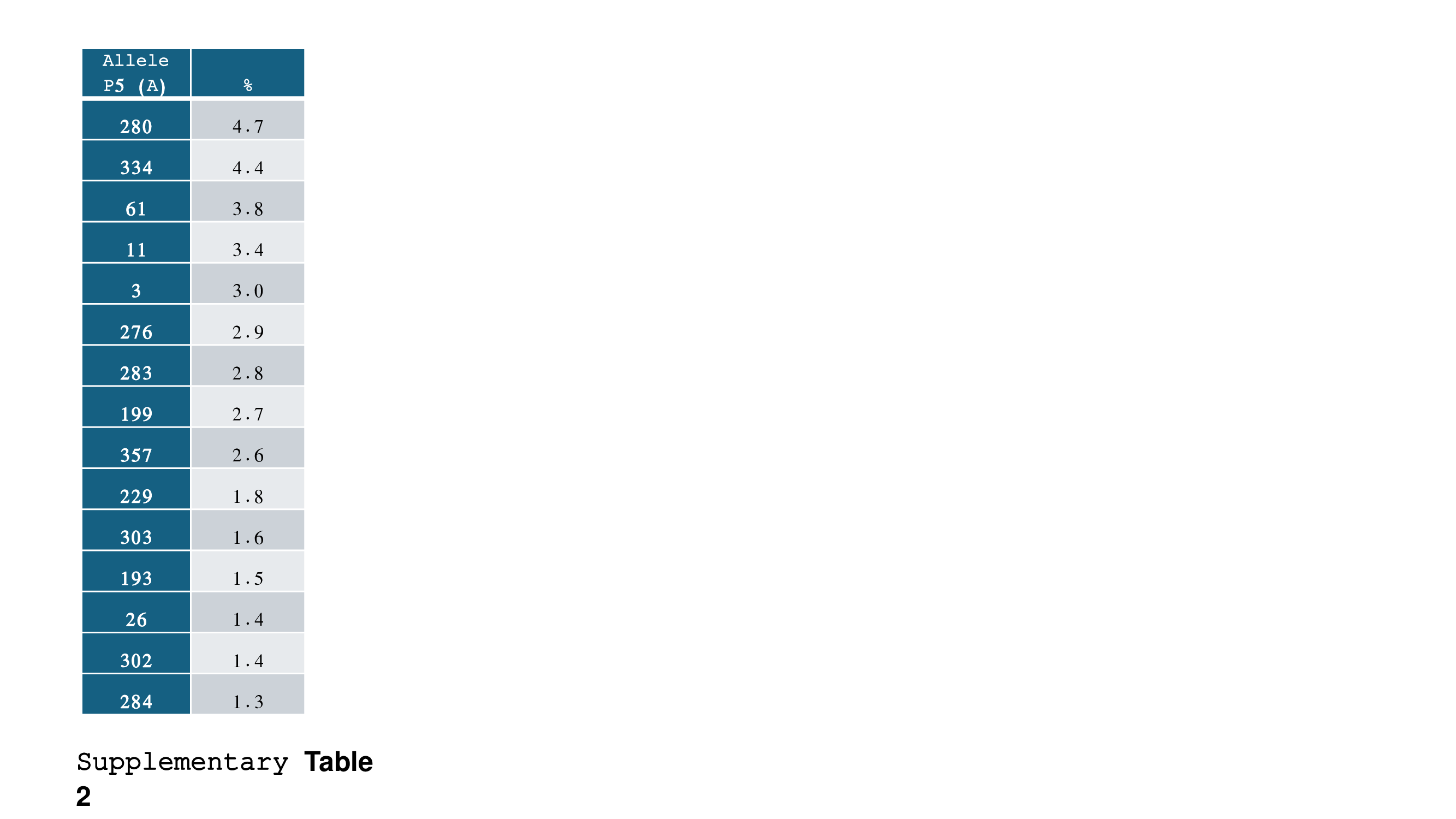

Supplement: Supplementary file 1 [file vaccines-13-00892-s001.zip › manuscript-supplementary/Supplementary-Table-2.tiff]

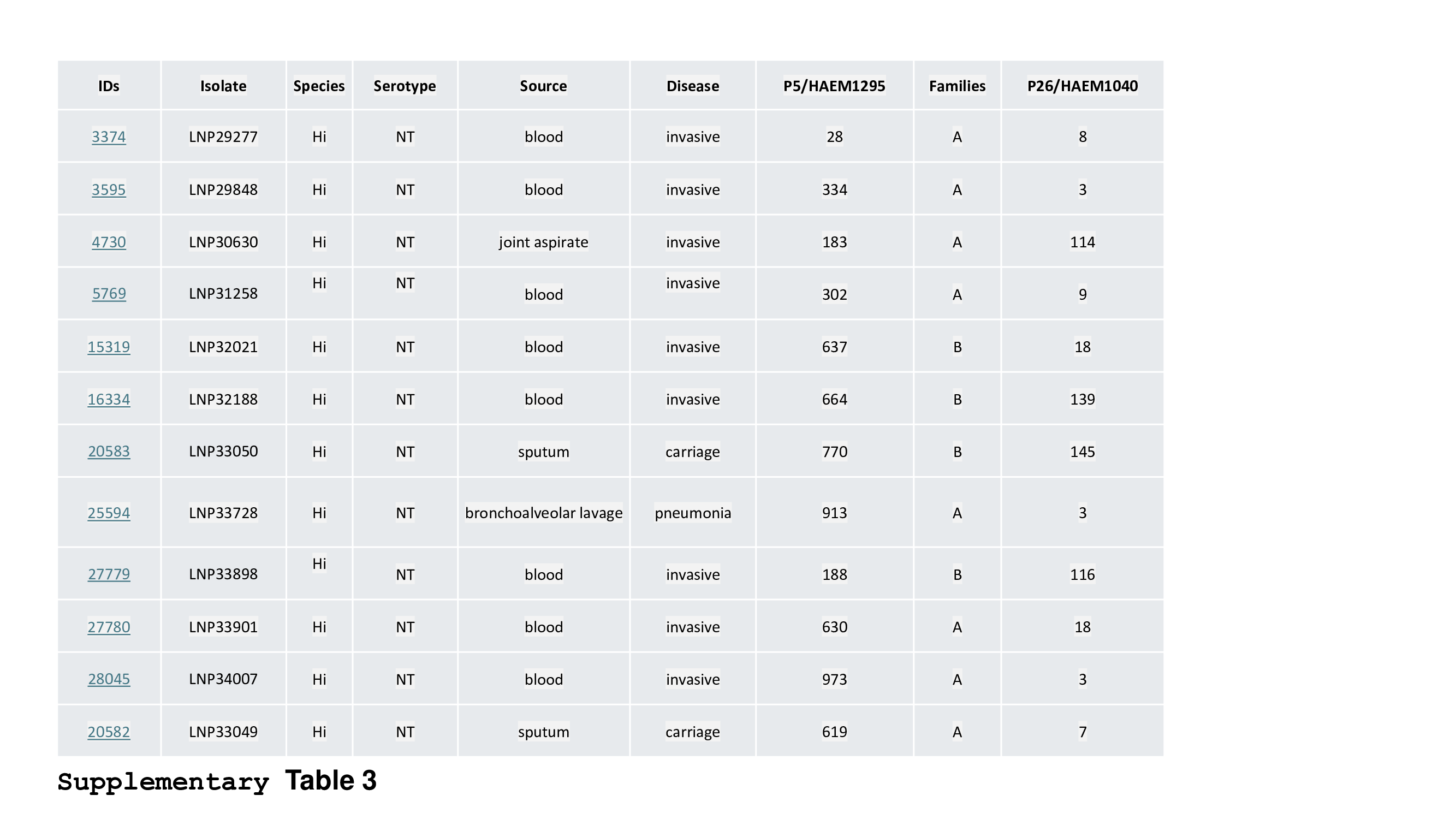

Supplement: Supplementary file 1 [file vaccines-13-00892-s001.zip › manuscript-supplementary/Supplementary-Table-3.tiff]

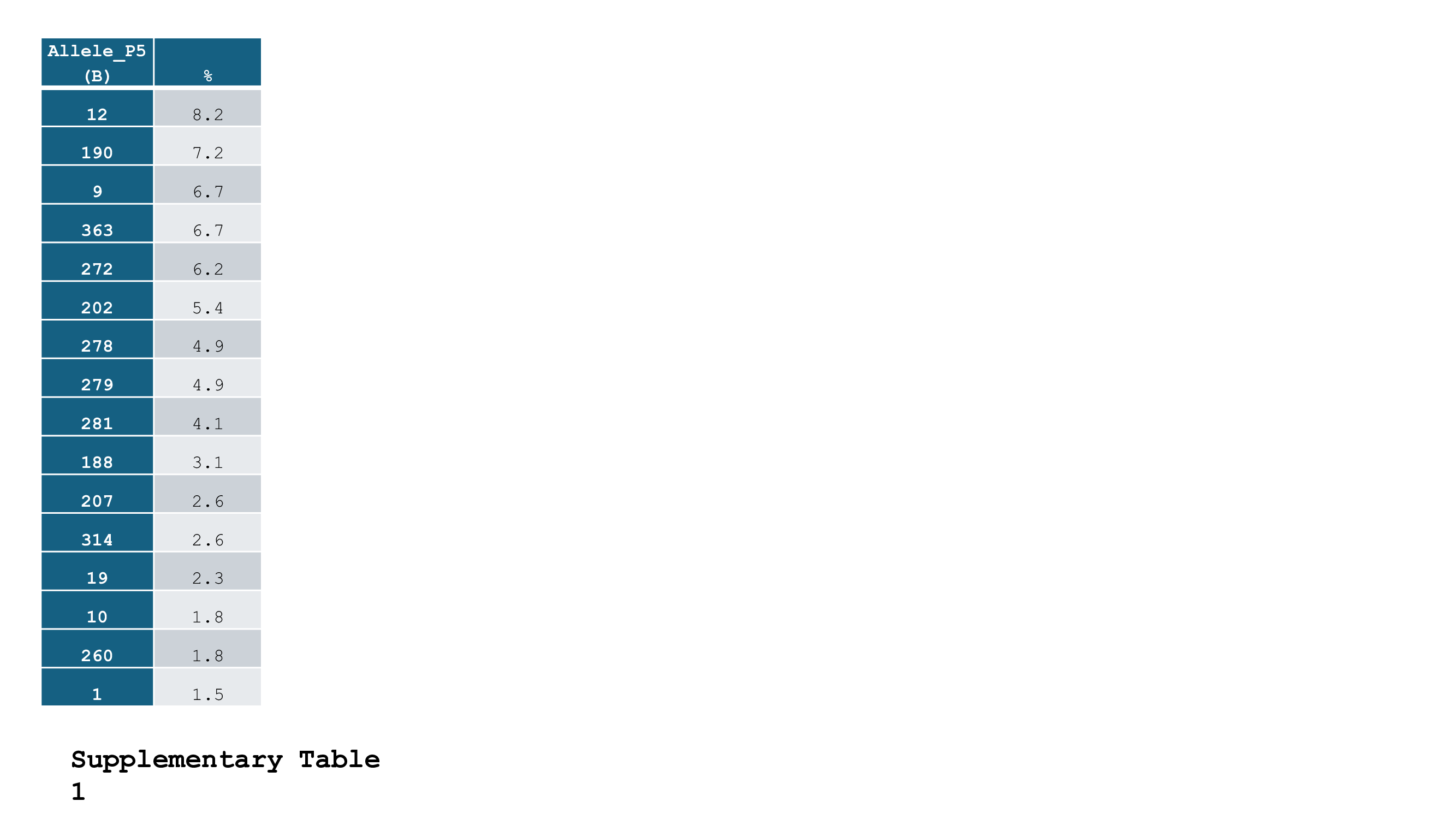

Supplement: Supplementary file 1 [file vaccines-13-00892-s001.zip › manuscript-supplementary/Supplementary-Table-1.tiff]
